# Molecular adsorbates as probes of the local properties of doped graphene

**DOI:** 10.1038/srep24796

**Published:** 2016-04-21

**Authors:** Van Dong Pham, Frédéric Joucken, Vincent Repain, Cyril Chacon, Amandine Bellec, Yann Girard, Sylvie Rousset, Robert Sporken, Maria Cristina dos Santos, Jérôme Lagoute

**Affiliations:** 1 MPQ, Université Paris Diderot-Paris 7, Sorbonne Paris Cité, CNRS, UMR 7162, 10, rue A. Domon et L. Duquet, 75205 Paris 13, France; 2 Research Center in Physics of Matter and Radiation (PMR), Université de Namur, 61 Rue de Bruxelles, 5000 Namur, Belgium; 3 Instituto de Física Universidade de São Paulo 05508-090, São Paulo, SP, Brazil

## Abstract

Graphene-based sensors are among the most promising of graphene’s applications. The ability to signal the presence of molecular species adsorbed on this atomically thin substrate has been explored from electric measurements to light scattering. Here we show that the adsorbed molecules can be used to sense graphene properties. The interaction of porphyrin molecules with nitrogen-doped graphene has been investigated using scanning tunneling microscopy and *ab initio* calculations. Molecular manipulation was used to reveal the surface below the adsorbed molecules allowing to achieve an atomic-scale measure of the interaction of molecules with doped graphene. The adsorbate’s frontier electronic states are downshifted in energy as the molecule approaches the doping site, with largest effect when the molecule sits over the nitrogen dopant. Theoretical calculations showed that, due to graphene’s high polarizability, the adsorption of porphyrin induces a charge rearrangement on the substrate similar to the image charges on a metal. This charge polarization is enhanced around nitrogen site, leading to an increased interaction of molecules with their image charges on graphene. Consequently, the molecular states are stabilized and shift to lower energies. These findings reveal the local variation of polarizability induced by nitrogen dopant opening new routes towards the electronic tuning of graphene.

The properties of graphene, arising from its two dimensional sp^2^ hybridized carbon lattice, make it an enticing material for developing novel carbon-based electronics and a formidable playground to explore fundamental solid state physics^1^. Graphene has been advantageously used in gas sensors^2^, single-molecule junctions^3^, transistors^4^ or supercapacitors^5^. Beyond the exploitation of bare graphene properties, chemical doping with heteroatoms can be used to tune the properties of graphene and improve device performances^6^,^7^,^8^,^9^,^10^,^11^,^12^. Nitrogen doping creates a local electronic modulation^13^ that can lead to active sites^14^,^15^ and enhances chemical reactivity and electrocatalysis reactions^16^. In such applications, the interaction of graphene with its surrounding environment is a central question. One driving force of such interactions is the polarizability of graphene that controls the electronic response of electrons in the presence of adsorbates or supporting substrates^17^. The charge modulation of graphene due to the interaction with adsorbed molecules having polar bonds has been studied^18^ and used to detect polar species in a graphene heterodyne sensor^19^. Tuning the polarizability of graphene appears therefore to be a cornerstone for controlling the interaction of graphene with its environment. For doped graphene, this requires an atomic scale study to investigate the local polarizability of graphene around doping sites.

Here, we report on scanning tunneling microscopy and spectroscopy (STM/STS) measurements and *ab initio* calculations of porphyrin adsorbed on nitrogen-doped graphene. Porphyrins belong to an important family of biomolecules and their properties at interfaces are of high current interest^20^ owing to the rich variety of supramolecular structures and nanomachinery/nanoelectronics promising applications already under investigation. In a previous work, we performed a comparative study of 5,10,15,20-Tetraphenyl-21H,23H-Porphyrin molecules (H_2_TPP) adsorbed on gold, graphene and nitrogen-doped graphene^21^. The porphyrins imaged by STM form large islands in graphene and N-doped graphene.

We found that the molecular resonances associated with the porphyrin frontier orbitals shifted to lower energy when the molecules were located nearby a nitrogen site leading to the appearance of bright molecules in the molecular islands. Using a statistical analysis of the density of nitrogen dopants and bright molecules we concluded that the effect of a nitrogen dopant extends over 0.8 nm leading to an average number of bright molecule 2.2 times larger than the number of nitrogen atoms. However a direct experimental observation of the location of nitrogen atoms below the molecular islands was still lacking. A theoretical interpretation based on *ab initio* calculations was still needed. In order to provide the lacking data and theoretical interpretation, we have used molecular manipulation to gain access to the surface below the molecules, thus revealing the link between the molecular states, energy shifts and the underlying nitrogen positions with unprecedented atomic scale precision. The results are interpreted with the aid of a density functional theory (DFT) molecular modeling of the adsorption of H_2_TPP on graphene and N-doped graphene flakes revealing that although a small charge transfer is found between the molecules and the graphene flake, a change of local polarizability around nitrogen can lead to the observed downshift of the molecular levels. DFT results are consistent with a binding mechanism involving charge polarization of graphene induced by the polar bonds of the porphyrin, such that the adsorbate interacts with its image charges on the substrate. The presence of nitrogen increases the local polarizability leading to an enhancement of the image charges and a stabilization (energy downshift) of the molecular states. This model reproduces the observed downshifts of the molecular resonances around a doping site and proves that nitrogen doping leads to a local increase of graphene’s polarizability over nanometer scale range. These findings show how heteroatomic doping can modify the polarizability of graphene opening new avenues to control the interaction of graphene with its environment.

We have deposited H_2_TPP molecules on nitrogen doped graphene following the procedure previously described^21^. Figure 1a shows a molecular island of H_2_TPPs on doped graphene. On the graphene area around the molecules, bright spots can be seen corresponding to the substitutional nitrogen atoms^13^,^22^. Inside the island, some molecules appear brighter. This is due to their proximity with nitrogen sites which induces a downshift of their HOMO (Highest Occupied Molecular Orbital) and LUMO (Lowest Unoccupied Molecular Orbital) states leading to a larger apparent height^21^.

In order to link the distribution of bright molecules with the location of underlying nitrogen sites, we performed molecular manipulation with the STM tip to remove the molecular island. This was achieved by scanning the whole image area with a setpoint turned to 0.2 V and 1 nA. We then turned back the setpoint to a regular imaging condition of 2 V and 50 pA. The image obtained after this operation is shown in Fig. 1b. As can be seen, nitrogen atoms in the previously covered area are revealed. By overlaying these N positions on molecular island before tip manipulation (Fig. 1a), the locations of underlying N with respect to the molecular positions are revealed.

With a high-resolution image on a smaller area as shown in Fig. 2, it is more straightforward to observe the relative N positions with respect to the bright molecules (see black dots on the molecular island). It is noticeable that the molecules centred above a N dopant are brighter (at bias voltage +2 V) whereas their nearest neighbours can display an intermediate level. Such a variation indicates that the presence of a nitrogen can modify the molecular state energies over a range that extends to several atomic sites. This is consistent with our previous measurements^21^ and can be linked to the extension of the density of states around the nitrogen atom that spreads over several atomic sites^13^ (see also supporting information).

This is illustrated with *dI*/*dV* spectra that have been recorded on different molecules as a function of the distance from N site. In Fig. 3 we show three spectra taken on three consecutive molecules as marked in the inset. The molecules in the inset show different contrasts: the molecule 3 is the highest one at 2 V, and molecule 2 appears with an intermediate height, while molecule 1 has the same height as the molecules far from N site which are not affected by a N dopant. The *dI*/*dV* spectrum taken on the molecule centred above N dopant (molecule 3) reveals HOMO and LUMO resonances at −1.53 V and 2.02 V (measured at the maxima of the peaks), respectively, which are lower than those of molecule 1 that appears at −1.32 V for HOMO and 2.22 V for LUMO. These values are fully consistent with those reported in our previous work^21^. However, the *dI*/*dV* spectrum recorded above molecule 2 shows intermediate energies at −1.38 V for HOMO and 2.09 V for LUMO. In order to gain more insight into this energy shift, we recorded *dI*/*dV* spectra as a function of the position along the lines crossing these molecules. The shift can be seen more clearly in the color spectra map shown in Fig. 3b where the shift of LUMO and HOMO energies are affected by the presence of an underlying N doping atom below the molecules, along a line in the inset of Fig. 3a. The molecule 3 centred on N atom, undergoes the largest energy shift with respect to the two other molecules 2 and 1, respectively, which are located further away from N site. The molecule 2 that lies just beside the molecule 3, is partly affected by N dopant and has a smaller energy shift as compared to molecule 3, while molecule 1 which is furthest from N, and is not affected by N dopant shows its molecular states at higher energies similar to the molecules that are far away from nitrogen atoms.

In order to gain further insight and rationalize the experimental findings, we performed *ab initio* calculations on model systems to understand the nature of the binding of porphyrin on graphene and the effects introduced by chemical doping. The graphene (G) model is a cluster including 286 carbon atoms built from a 12 × 12 graphene unit cells and saturated by hydrogen at the zigzag edges. In the N-doped graphene (NG) model two carbon atoms from G model, one at the cluster centre and the other near one corner, were replaced by nitrogen atoms.

The ground state geometries of G and NG are planar and C-C bond lengths average to 1.425 Å, while C-N bonds are shorter (1.410 Å). The most important difference between these clusters is the charge distribution: the two substitutional nitrogen atoms in NG add two extra electrons into the *π* system, thus raising the Fermi energy (or the charge-neutrality energy) by 0.08 eV with respect to that of G. The combined effect of extra electrons and the presence of the polar C-N bonds is a charge oscillation that extends over a radius of ≈8 Å from the substitutional site, as illustrated in Fig. 4. This figure shows the charge density difference between NG and G as an isosurface of 0.0003 e/bohr, with cyan (gold) colour representing increase (depletion) of electronic density. We placed one of the doping sites near the cluster edge, where a small charge polarization due to C-H bonds is already present, resulting in an amplification of the N-induced charge oscillation, but it is nevertheless separated from the effects due to the central doping site.

Adsorption of H_2_TPP on G and on NG resulted in very similar geometries. In both cases the adsorbed porphyrin adopts a saddle-shaped geometry with its centre lying above a C-C bond of the substrate, at distances 3.47 and 3.41 Å, respectively, from G and NG. The dopant site of NG is between two neighboring nitrogen atoms of porphyrin. The phenyl ligands torsion angles decrease, from 58 in free H_2_TPP to 47 in the adsorbed state. Charge transfer toward the macrocycle is very small in both substrates, amounting to 0.06–0.07 of an electron (from Mulliken population analysis) which indicates that the molecule is physisorbed. This small amount of charge transfer is consistent with the large energy distance between the LUMO state and the Fermi level preventing from a transfer of a full electron that would bring the LUMO state at the Fermi level. The binding energy is thus due to dispersion and to the electrostatic interaction coming from the charge redistribution on the substrates induced by the polar bonds of H_2_TPP. Dispersion contributions to the binding are nearly the same in G and NG, as expected. However, as the larger molecule-substrate distance of the G cluster indicates, the binding energy of the H_2_TPP-NG system is stronger by 0.14 eV and the origin of this difference is the increased electrostatic interaction between the formal charges on porphyrin atoms and their image charges on the substrate.

The static dipole polarizability *α* of a system can be written, according to the charge-dipole model^23^, as:


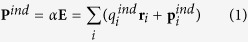


where **P**^*ind*^ is the induced dipole moment due to an external field **E**. In the above equation the induced polarization is expressed as a sum of the atomic contributions, namely, the induced charge 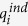 of the atom located at *r*_*i*_ and its induced dipole moment 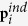. Local variations of polarizability can thus be related to the variations of induced charges under the external field. The induced charges can be calculated through density differences obtained in *ab initio* calculations and, as we show below, the induced charges on NG due to the electric field of the adsorbed porphyrin are larger than those induced on G. This means that the local polarizability of NG around the doping site is larger than that of pristine graphene and it extends further away from the doping site since adsorbed molecules nearby the doping site are also affected by a stronger electrostatic interaction.

Figure 5 displays the electronic density difference in the substrate, namely, the difference between the graphene electronic densities before and after adsorption of H_2_TPP. A and B structures correspond to the lowest energy conformation of the macrocycle adsorbed on G and on NG, respectively. Notice the gold surface underneath the four nitrogen atoms of the macrocycle, which means an accumulation of positive charge on the substrate in response to the net negative charge of the nitrogen atoms above it. Similarly, an accumulation of negative charge occurs underneath the four phenyl ligands that point to the substrate and place the positively charged hydrogen atoms in close proximity to the substrate. The larger extension of the charge redistribution of B when compared to A translates to larger image charges. The panels below A and B figures show a side view including porphyrin density differences. The depletion of electronic density above graphene indicates that no covalent bonding occurs between the molecule and graphene and that the interaction is of electrostatic nature. We conclude that NG is locally more polarizable than G. Structures C and D were obtained by translating the macrocycle along the NG substrate without optimizing the geometry, but keeping the distance of the central part of the molecule at 3.41 Å from NG. In C the porphyrin is between two doping sites while in D its central ring is all above carbon sites but it is still close to the doping site at the center. These structures suffer the influence of the cluster edge bonds through the phenyl ligands, giving rise to larger regions of induced negative charges, however due to charge neutrality the positive charges are present in same amounts but spread differently from A and B.

We now turn to the analysis of the electronic structures. Figure 6 shows the total density of states (DOS) and the density of states projected (PDOS) on H_2_TPP. In Fig. 6a we show the DOS of the combined G-H_2_TPP system and the PDOS with respect to the cluster Fermi energy. The resonances corresponding to the molecular HOMO and LUMO levels are located at −0.93 eV and 1.57 eV, respectively, resulting in a gap of 2.50 eV. The experimentally measured values are −1.34 eV and 2.00 eV so that there is an almost symmetrical mismatch of ≈0.4 eV between theory and experiment that is due to a well known difficulty of Kohn-Sham eigenvalues of DFT theory in reproducing fundamental gaps^24^. For instance, the absolute values of HOMO and LUMO states of free H_2_TPP are, within the B3LYP (see Methods) calculation, −4.86 eV and −2.22 eV, respectively, with a resulting gap of 2.64 eV. We calculated the total energy of positive and negative ions to obtain the so-called ΔSCF (difference of total energies) estimation of ionization energy and electron affinity, which are 5.98 eV and 1.06 eV, respectively. Hence, the ΔSCF fundamental gap is 4.92 eV, in much better agreement with the value of 4.63 eV obtained from experimental ionization energy^25^ and electron affinity^26^. Several methods have been proposed to correct for this deficiency of DFT in the calculation of fundamental gaps, including the GW method^27^ and the image charges method^28^,^29^,^30^. We will not attempt to correct our DFT results since the calculated Kohn-Sham eigenvalues are not far from the experimental resonances and because the effects we want to explain from calculations apparently do not depend on this correction. As a test for the image charge effect on the molecular levels of porphyrin, we performed a calculation of free porphyrin interacting with a collection of point charges placed at the mirror images of the net atomic charges of the molecule, assuming a porphyrin-mirror distance of 3.4 Å. We used the Mulliken atomic charges of the free molecule for the image point charges. The frontier Kohn-Sham eigenvalues lowered uniformly by 0.13 eV with respect to the free molecule, thus justifying the above arguments.

Figure 6b shows the DOS and PDOS on NG. As stated earlier, NG Fermi energy is 0.08 eV above the Dirac point. The HOMO and LUMO resonances are located at −1.24 eV and 1.25 eV, respectively. Part of the shift to lower energies when compared with Fig. 6a is explained by the Fermi energy shift but there is still a lowering of 0.24 eV in both resonances to be explained. The stabilization of the molecular frontier orbitals is due to the interaction with the increased image charges induced on NG when compared to G. This is further corroborated by the results displayed in Fig. 6c, which compares the PDOS obtained for the configurations shown in Fig. 5b–d. The curves in Fig. 6c are labeled accordingly. When moving the porphyrin from the position B (lowest energy conformation) to position C (between two doping sites), the local polarizability decreases, and the positive image charges underneath the center of the macrocycle decreases, pushing the resonances to higher energies. In configuration D the center of the molecule is on carbon sites but it is still close enough to the doping site to experiment the effects of the higher polarizability provoked by nitrogen doping. As a consequence, the resonances slightly shift to higher energies. This is in agreement with the experimental findings. We conclude that the porphyrin adsorption on nitrogen-doped graphene not only signals where the doping sites are located through the level shifting that produces the bright molecules in the STM images, but also they show the extension of polarizability changes induced by the dopant. The level shifting of adsorbates is probably not an unique property of doping sites and we could propose, based on our findings, that other types of graphene defects could be visible through the effects on an adsorbate.

In summary, combining STM/STS with DFT calculations we showed that nitrogen doping of graphene leads to a local enhancement of graphene polarizability. This enhancement was probed by measuring the energy shift of HOMO and LUMO states of molecular adsorbates. These shifts are due to the interaction between the molecules and their image charges. The larger is the local polarizability, the larger the image charges and the larger is the HOMO/LUMO downshift. The nitrogen atoms induce variations of the polarizability over a distance that spreads to several atomic sites and, as a consequence, depending on the respective molecule-nitrogen locations, one nitrogen atom can influence more than one molecule. Our findings show that nitrogen doping can be used to modify the polarizability of graphene and how this polarizability is modified with atomic scale precision.

## Methods

### Experimental

The experiments were carried out using an Omicron LT-STM operating at ≈4.6 K under UHV condition with a pressure lower than 1 × 10^−10^ mbar. All the STM and STS measurements reported here were obtained at 4.6 K. The *dI*/*dV* spectra were acquired using a lock-in technique at a frequency of ca. 670 Hz and a modulation amplitude of 35 mV. The measurements were performed with an electrochemically etched tungsten tip. Graphene samples containing multilayer (≈5–10) were obtained on SiC(000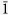) by annealing the substrates in ultrahigh vacuum (UHV) at 1320 °C for 12 min under a silicon flux of ≈1 ML/min. The doping of nitrogen into graphene was obtained by exposing the graphene sample to a flux of nitrogen radicals produced by a remote radio-frequency plasma source^13^. Pristine and nitrogen-doped graphene samples were taken out of the UHV synthesis chamber and transferred in air to another UHV system for low temperature STM measurements. Then, the samples were degassed at ≈800 °C for few minutes before the measurements. Free base tetraphenylporphyrin (H_2_TPP) molecules (Aldrich) were deposited using an effusion cell (Dr. Eberl MBE-Komponenten GmbH) under vacuum at 255 °C onto the samples at the STM stage maintained at 4.6 K. The graphene samples were then brought to room temperature in few hours, allowing molecules to self-assemble and form a 2D island on the surface.

### Computational Details

Electronic structure calculations of the present study were performed in the framework of Density Functional Theory (DFT). The hybrid B3LYP functional^31^ (Becke, three-parameter, Lee-Yang-Parr exchange-correlation functional) with empirical van der Waals correction^32^ and a gaussian 6–31(d,p) basis set was employed. The geometry of H_2_TPP molecule was optimized in three distinct situations: (i) isolated molecule, (ii) molecule adsorbed on graphene, and (iii) molecule adsorbed on nitrogen-doped graphene. Graphene (G) was modeled as 12 × 12 unit cells graphene cluster (286 carbon atoms) saturated with hydrogen at the edges. Nitrogen-doped graphene (NG) was built by substituting two carbon atoms (21.3 Å apart) by nitrogens, thus representing a doping level of 0.7%. The cluster geometries were also optimized prior to the adsorption of H_2_TPP for the analyses of charge distributions and adsorption energy calculations. Total densities of states (DOS) and projected densities of states (PDOS) were calculated by adopting a gaussian broadening (0.2 eV) of Kohn-Sham eigenvalues. DFT calculations were carried out within Gaussian 09 package^33^ and post-processing data were obtained with Multiwfn^34^ and Chimera^35^ packages.

## Additional Information

**How to cite this article**: Pham, V. D. *et al.* Molecular adsorbates as probes of the local properties of doped graphene. *Sci. Rep.* **6**, 24796; doi: 10.1038/srep24796 (2016).

## Supplementary Material

Supplementary Information

## Figures and Tables

**Figure 1:**
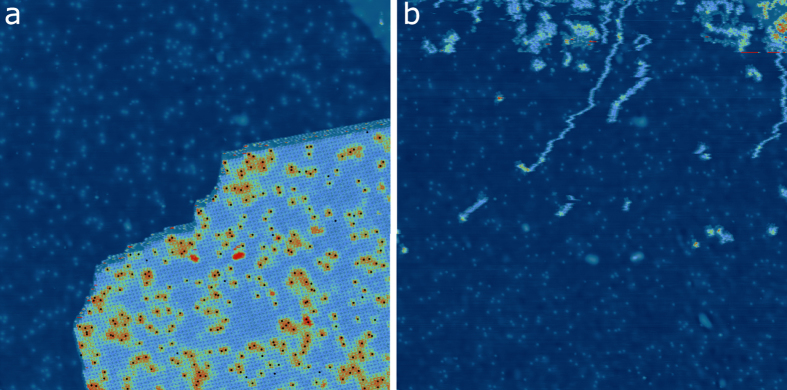
(**a**) STM images (150 × 150 nm^2^, at 2 V, 50 pA) of a molecular island before (**a**) and after (**b**) tip manipulation. The black dots in (**a**) indicate the position of the nitrogen atoms below the molecular island extracted from (**b**).

**Figure 2:**
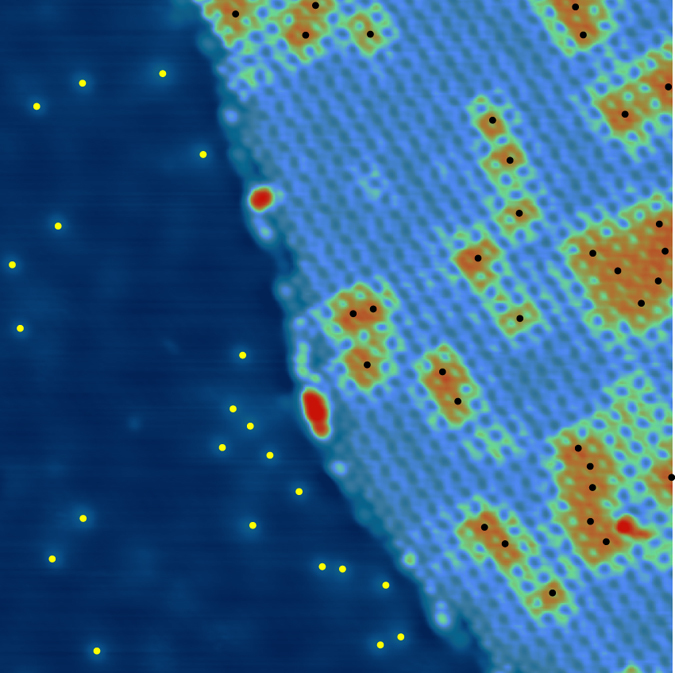
STM image (50 × 50 nm^2^, 2 V, 100 pA) with superimposed nitrogen positions (black dots on molecular island and yellow dots on graphene area). Small scale imaging reveals that one nitrogen atom can affect more than one H_2_TPP molecule.

**Figure 3:**
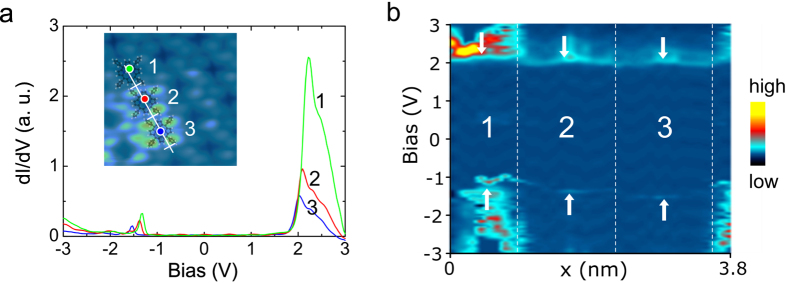
(**a**) *dI*/*dV* spectra measured on three adjacent molecules around a nitrogen site. Inset: high-resolution STM image (5 × 5 nm^2^, 2 V, 200 pA) with markers indicating the molecules where the spectra have been measured and molecular models to guide the eyes. (**b**) Color map representing the *dI*/*dV* spectra measured over the line marked in the inset in (**a**), which shows different shifts of the molecular states on the three molecules marked in (**a**). The vertical dashed lines correspond to the positions indicated by the three marks on the line in the inset of (**a**). The arrows indicate the traces corresponding to HOMO (bottom arrows) and LUMO (top arrows) states of the three molecules.

**Figure 4:**
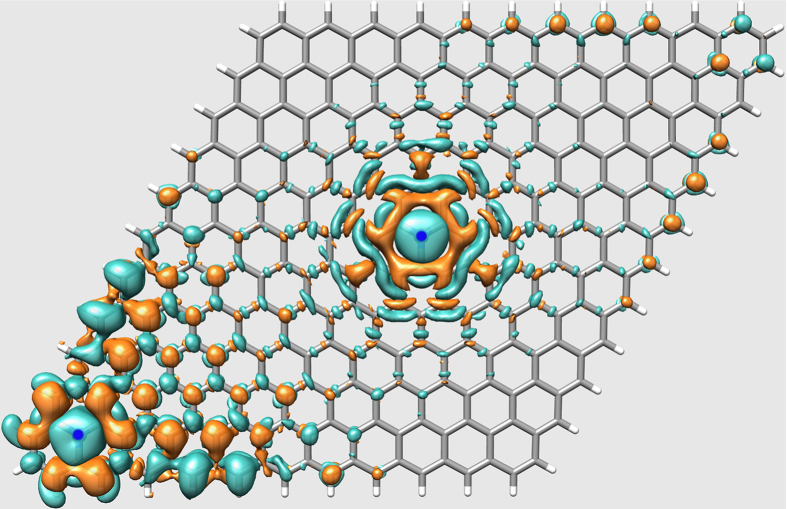
Electronic density difference between graphene and N-doped graphene. The cyan isosurface (0.0003 e/bohr^3^) represents an increase and the golden isosurface (−0.0003 e/bohr^3^) represents a decrease of electronic density. Color scheme: carbon is gray, hydrogen is white, and the blue dots mark the position of nitrogen atoms.

**Figure 5:**
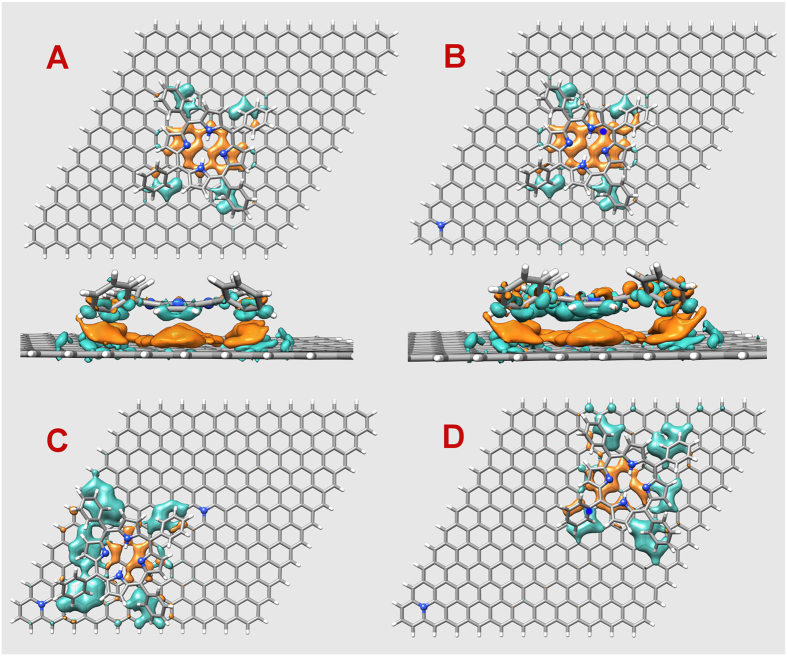
Charge redistribution due to adsorption of H_2_TPP on graphene. (**A**) lowest energy conformation on pristine graphene (side view on lower panel). (**B**) N-doped graphene, lowest energy conformation where the macrocycle sits over the doping site (side view on lower panel). (**C**) the porphyrin is between the two doping sites. (**D**) the porphyrin is near the doping site but the central ring sits over carbon sites. The isosurfaces (0.00025 e/bohr^3^) of gold color represent density depletion and those of cyan color represent increased electron density. Color scheme: carbon is gray, hydrogen is white, nitrogen is blue, and the blue dots in (**B**,**D**) mark the position of nitrogen atoms.

**Figure 6:**
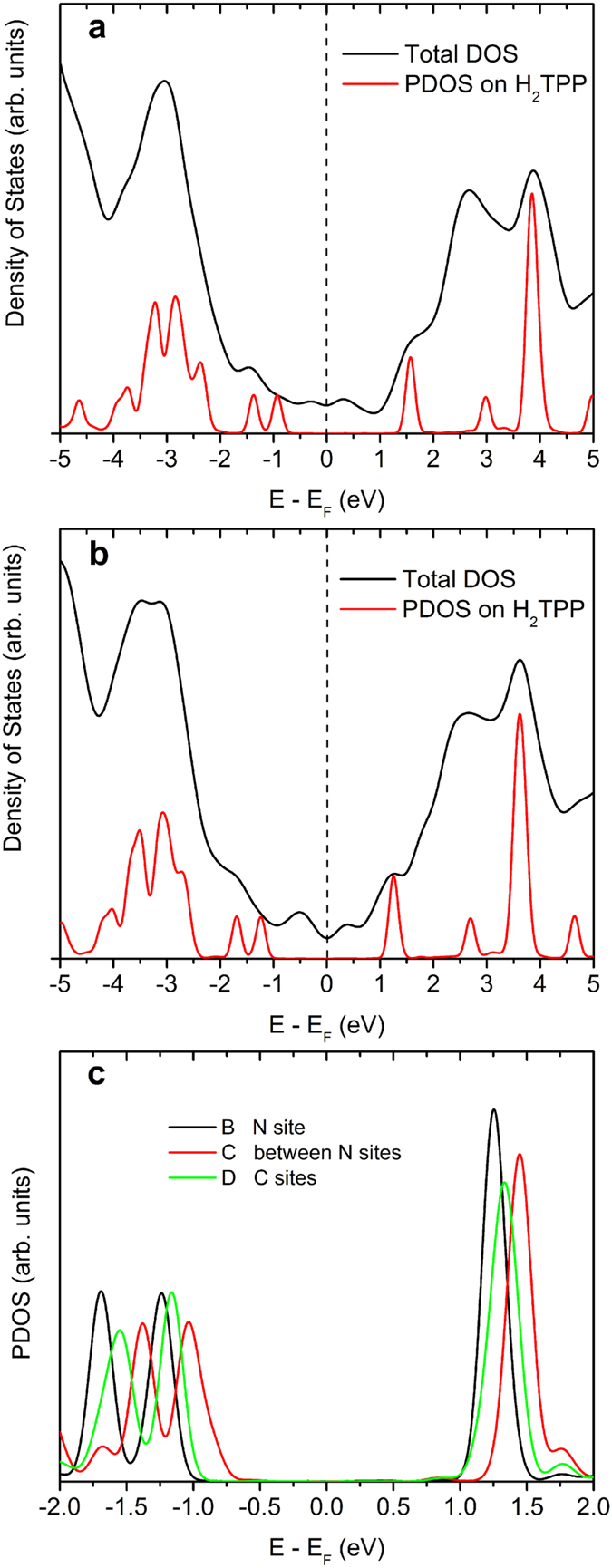
Density of states (DOS) and porphyrin-projected density of states (PDOS). (**a**) H_2_TPP on graphene. (**b**) H_2_TPP on nitrogen-doped graphene. (**c**) PDOS for structures (**B**–**D**) of Fig. 4, showing the adsorption position level shifting.
