# Supplementary material for: Molecular adsorbates as probes of the local properties of doped graphene
Source: Sci Rep. 2016 Apr 21;6:24796. doi: 10.1038/srep24796 (PMC4838864; doi:10.1038/srep24796)
Supplement: Supplementary Information [file srep24796-s1.pdf]

Supporting information for: Molecular adsorbates as probes of the local properties of doped graphene

Van Dong Pham[1], Frédéric Joucken[2], Vincent Repain[1], Cyril Chacon[1], Amandine Bellec[1], Yann Girard[1], Sylvie Rousset[1], Robert Sporken[2], Maria Cristina dos Santos[3], and Jérôme Lagoute[1]

[1] *MPQ, Université Paris Diderot-Paris 7, Sorbonne Paris Cité, CNRS, UMR 7162, 10, rue A. Domon et L. Duquet, 75205 Paris 13, France.* [2]*Research Center in Physics of Matter and Radiation (PMR), Université de Namur, 61 Rue de Bruxelles, 5000 Namur, Belgium.* [3]*Instituto de Física Universidade de São Paulo 05508-090, São Paulo, SP, Brazil*

E-mail:

In Fig. S1, we show an area of nitrogen doped graphene (Fig. 1a) revealing a few substitutional nitrogen atoms. On that area, we have recorded a dI/dV map simultaneously with the topography (Fig. S1b). As previously reported, a localized electronic state appears around the nitrogen atoms.[1] The dI/dV map reveals the spatial extent of this localized state around the nitrogen atoms. This state corresponds to a peak at positive bias observed in dI/dV spectra (see Fig. S1c).

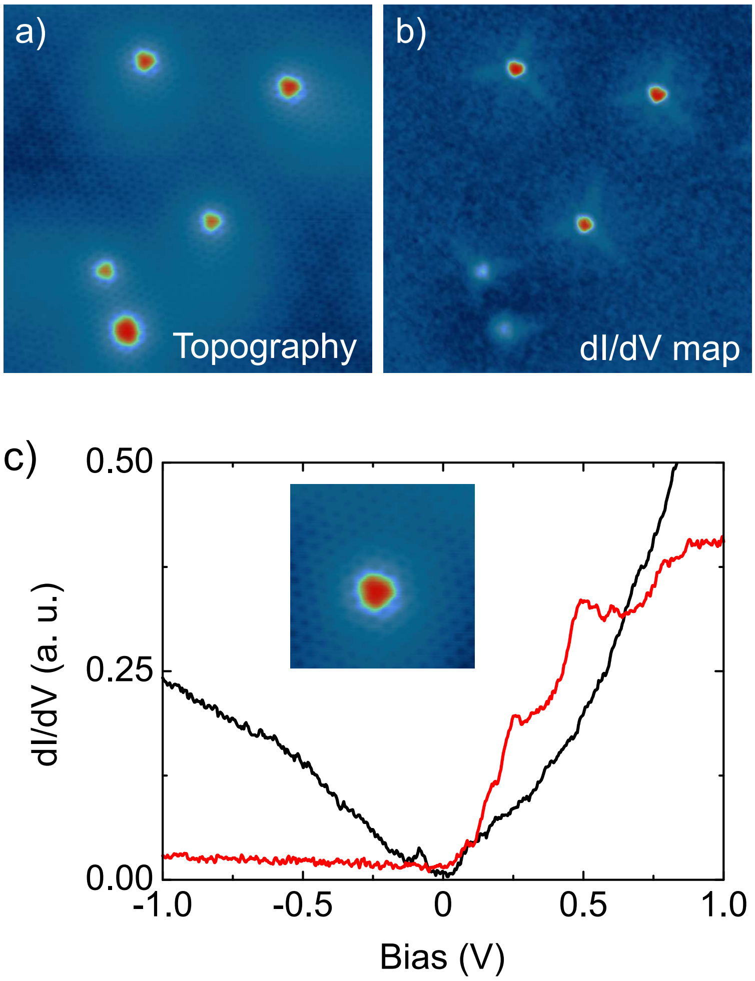

Figure S 1: (a) Topographic STM image of nitrogen doped graphene (10×10 nm^2, at 2 V, 500 pA). (b) dI/dV map at 0.54 V recorded simultaneously with image in (a). (c) dI/dV spectra measured on graphene (black curve) and above the nitrogen atom (red curve) shown in the inset (zoom of (a), 3×3 nm^2).

References

1. Joucken F. *et al.* Localized state and charge transfer in nitrogen-doped graphene. *Phys. Rev. B* **85**, 161408 (2012).
